# Relationship Between Serum Vitamins and Cognitive Impairment in the Elderly: A Study Based on the NHANES Database

**DOI:** 10.1002/brb3.71181

**Published:** 2026-01-13

**Authors:** Jiarui Miao, Danyu Zhao

**Affiliations:** ^1^ Basic Medical College Liaoning University of Traditional Chinese Medicine Shenyang Liaoning China

**Keywords:** cognitive impairment, folic acid, mixed exposure, vitamin B12, vitamin D

## Abstract

**Background:**

Vitamins, as a modifiable lifestyle factor, are increasingly recognized for their protective role in cognitive health. However, the synergy or interaction among vitamins remains unclear.

**Objectives:**

This work aimed to analyze the association of serum vitamin D, folic acid (FA), and vitamin B12 with cognitive impairment in the elderly.

**Methods:**

Data included 2582 elderly participants aged 60 and older from the NHANES, 2011–2014. Weighted logistic regression, Bayesian kernel machine regression (BKMR), and weighted quantile sum (WQS) models were used to analyze the association of serum vitamins and their mixture with cognitive disorder risk. Interaction effects among vitamins were investigated. Sensitivity analyses accounting for vitamin supplements, depression, and sleep disorders, and subgroup analyses focusing on high vitamin B12 levels were performed.

**Results:**

After adjusting for confounding factors, serum Vitamin D (OR = 0.695, 95% CI: 0.534–0.905, *p *= 0.003) and FA (OR = 0.777, 95% CI: 0.604–0.999, *p *= 0.034) levels were inversely correlated with cognitive disorder risk. Both remained robust after considering vitamin supplements and comorbidities in the sensitivity analyses. The BKMR model indicated a significant increase in cognitive impairment risk when the overall vitamin mixture level fell below the 50th percentile. A U‐shaped association was detected between vitamin B12 and cognitive disorder risk. Vitamin B12 and FA had potential interaction effects. The WQS model revealed the largest contribution by FA (56.0%) to the overall protective effect on the cognitive disorder risk. The association with high vitamin B12 was predominantly observed in individuals with specific metabolic conditions, including kidney stones and hypertension.

**Conclusion:**

Optimizing vitamin D and FA levels remains the key to reducing cognitive impairment risk in FA‐supplemented populations. Vitamin B12 management requires greater precision; its high level in specific metabolic patients may signal health risks. This study provides new evidence for precise nutritional intervention for the elderly.

## Graphical abstract

This cross‐sectional study of 2582 older adults investigates the associations of serum vitamins D, B12, and folate with cognitive impairment. We found vitamin D and folate were inversely associated with risk, while vitamin B12 exhibited a U‐shaped relationship. High vitamin B12 risk was concentrated in individuals with metabolic comorbidities, suggesting the need for precise nutritional management beyond universal supplementation.

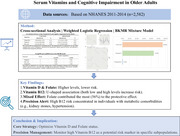

## 1 Introduction

With the accelerated process of population aging, the incidence of cognitive impairment in the elderly continues to increase (Perez Palmer et al. 2022). Mild cognitive impairment is defined by a significant decline in one or more cognitive domains in neurocognitive testing, which is not sufficient to interfere with independence (particularly in basic self‐care activities) or develop into dementia (Winblad et al. 2004, Burns et al. 2023). Without timely intervention, some patients may gradually develop dementia, which seriously affects the activity of daily living and quality of life (McGrattan et al. 2022). According to statistics, the number of dementia patients worldwide was approximately 57.4 million in 2019, and it is projected to reach 152.8 million in 2025 (GBD 2019 Dementia Forecasting Collaborators 2022), which will pose a daunting challenge to family care, medical resources, and the socio‐economic system (S. Chen et al. 2024). Therefore, the identification of risk factors that can be intervened in and the adoption of effective early prevention measures are of great significance for alleviating cognitive impairment and lowering the risk of dementia.

The function of vitamin levels in cognitive function maintenance as a preventable lifestyle factor has attracted much attention. The supplementation of Group B vitamins is linked with decreased homocysteine levels, and hyperhomocysteinemia is considered to be an independent risk factor for cognitive decline and the risk of dementia (Olaso‐Gonzalez et al. 2022, Nie et al. 2014). A VITACOG intervention study targeting individuals with mild cognitive impairment showed that supplementing Group B vitamins can effectively slow down the process of brain atrophy in patients by reducing homocysteine levels (Smith et al. 2010). Furthermore, a randomized, single‐blind, placebo‐controlled trial showed that the supplementation of folic acid (FA) and vitamin B12 has beneficial therapeutic effects on patients with Alzheimer's disease without an FA‐fortified diet (H. Chen et al. 2021). Vitamin D is instrumental for bone metabolism and also has potential value in brain and neural protection (Makris et al. 2021, Cui and Eyles 2022). Low vitamin D is proven to be linked with dementia, Alzheimer's disease, and cognitive impairment (X. X. Zhang et al. 2024). However, there is a study showing that vitamin D supplementation does not considerably enhance cognitive ability (Montero‐Odasso et al. 2023). Although the relationship between single vitamin levels and cognitive function has been analyzed separately, in the actual population, multiple vitamins participate in a series of physiological processes together, and there may be synergy or interaction among them. If the potential interaction is ignored, the true impact of vitamins on cognitive health may be underestimated.

Therefore, based on the National Health and Nutrition Examination Survey (NHANES) database, this work aimed to analyze the correlation of three key serum vitamins (vitamin B12, vitamin D, and FA) and their mixtures with cognitive impairment in the elderly, trying to offer a scientific basis for the early identification and nutritional intervention of cognitive impairment in the elderly and theoretical support for the formulation of nutrition‐based prevention strategies.

## 2 Methods

### 2.1 Study Population

**FIGURE 1:**
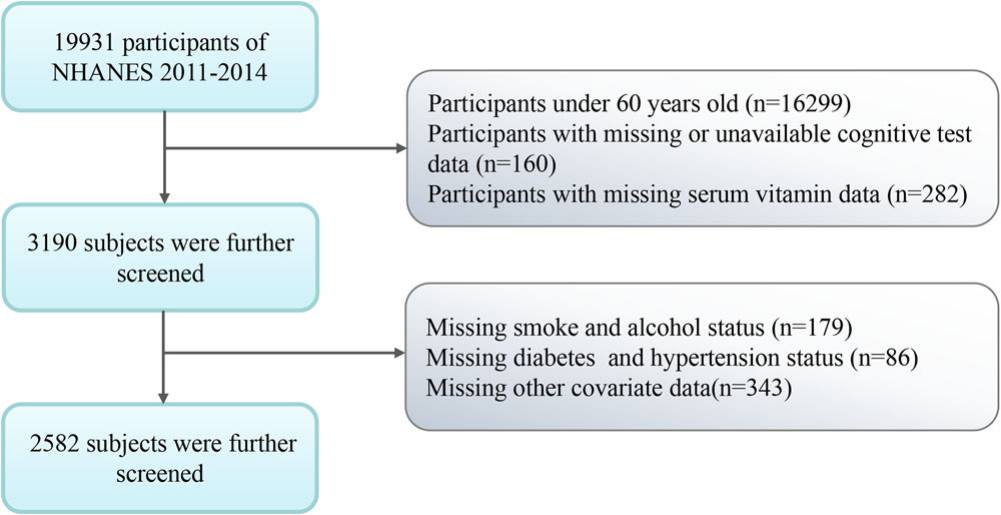
Flowchart of screening NHANES participants.

NHANES is a comprehensive study conducted by the Centers for Disease Control and Prevention (CDCP) and the National Center for Health Statistics (NCHS) to examine the health and nutritional status of the non‐institutionalized American population through stratified and multistage sampling methods. This study utilized NHANES data from two survey cycles from 2011 to 2014 (*n* = 19,931). After excluding participants < 60 years of age (*n* = 16,299), participants with missing or invalid cognitive test data (*n* = 160), participants with missing or invalid serum vitamin data (*n* = 282), and participants with other missing covariate data (*n* = 608), a total of 2582 people (Figure 1) were enrolled in our analysis.

### 2.2 Independent Variable

The blood samples of the participants were collected at the Mobile Examination Centre. Serum vitamin B12 levels were measured by the Roche Elecsys 170 automatic electrochemiluminescent immunoassay. Serum total FA levels were examined by high‐performance liquid chromatography‐tandem mass spectrometry (HPLC‐MS/MS). Serum levels of 25‐hydroxy (25(OH))‐vitamin D2 and 25(OH)‐vitamin D3 were determined by ultra‐high performance liquid chromatography‐tandem mass spectrometry (UHPLC‐MS/MS). The vitamin D level in this study was the sum of the two.

### 2.3 Dependent Variable

Three cognitive tests were conducted to determine cognitive function in participants, including the Animal Naming Test, the Consortium to Establish a Registry for Alzheimer's Disease Registry for Word Learning subtest (CERAD W‐L), and the Digit Symbol Substitution Test (DSST). CERAD W‐L is applied to assess people's immediate and delayed learning capability of new vocabulary information (Morris et al. 1989), including three consecutive learning trials and one delayed recall trial. In the learning experiment, participants were supposed to read 10 unrelated words aloud and recall as many words as possible quickly after the words were displayed. The order of words in each trial was different, and the highest score for each round of trials was 10 points. Delayed recall was undertaken after the Animal Naming Test and DSST. The maximum total score for the CERAD W‐L section was 40 points.

The Animal Naming Test was utilized to assess participants’ fluency when speaking languages of different categories (Morris et al. 1989). Participants were supposed to speak out as many animal names as possible within a minute, with 1 point scored for each name.

DSST was applied in evaluating sustained attention, processing speed, and working memory. The test was undertaken by utilizing a paper questionnaire with nine numbers and their corresponding symbols listed at the top of the questionnaire. Participants were required to fill in the corresponding symbols in the 133 spaces below according to the matching rules of numbers and symbols within 2 min. The total number of tests scored as correct pairs.

Based on three cognitive tests, the *z*‐scores of each test were calculated based on the average value (*μ*) and standard deviation (*σ*) of each test. The formula is *z*‐score = (*x −* *μ*)/*σ*, where *x* represents the original score of the participants in the test. The *z*‐scores of the three tests were then summed to obtain an overall cognitive score (Shi et al. 2023). Since there is no uniform diagnostic standard for cognitive disorders, we reviewed existing research methods and identified individuals with an overall cognitive score lower than the minimum quartile as cognitively disordered individuals in this study, with the corresponding classification threshold set as –1.632.

### 2.4 Covariates

The covariables in this study included Body Mass Index (BMI), gender, age, smoking, educational levels, drinking, hypertension, diabetes, physical activity (PA), total cholesterol, HDL‐C, vitamin supplements, depression, sleep disorders, anemia, kidney stones, and hyperuricemia. (1) Participants were sorted into three groups according to BMI: < 25 kg/m^2^, 25–30 kg/m^2^, and > 30 kg/m^2^ (Tian et al. 2022). (2) Participants were divided into three categories according to their smoking habits: never smoking, past smoking, and current smoking (previous smokers: people who smoked more than 100 cigarettes in their lifetime but no longer smoke; never smokers: people who smoked less than 100 cigarettes in their lifetime; current smokers: people who smoked more than 100 cigarettes in their lifetime and are still smoking) (Y. Zhang et al. 2022). (3) Alcohol consumption was defined as drinking at least 12 drinks per year (Dong et al. 2020). (4) Whether the participant suffered from hypertension was determined according to any of the following criteria: (1) the participant was informed of hypertension by a doctor or other health professional; (2) the participant took prescription drugs for lowering blood pressure; (3) the participant had diastolic pressure ≥ 80 mmHg or shrinkage pressure ≥ 130 mmHg (Liang et al. 2022). (5) The diagnostic basis for diabetes included any of the following conditions: (1) being diagnosed with diabetes by a doctor or health professional; (2) glycosylated hemoglobin level ≥ 6.5%; (3) fasting blood glucose ≥ 126 cm; (4) taking hypoglycemic drugs (Zhao and Li 2022). (6) Three groups were divided in terms of PA: the none‐PA group, the moderate‐PA group, and the intense‐PA group. Moderate PA was defined as tasks that caused mild sweating or a slight increase in breathing or heart rate. Intense‐PA was defined as tasks that cause massive sweating or a dramatic increase in breathing or heart rate. (7) The use of vitamin supplements was defined as taking dietary supplements containing vitamin D, FA, or vitamin B12 within the past 30 days, based on data from the NHANES Dietary Supplement Questionnaire (DSQ). Specifically, if participants reported a total intake of vitamin D (D2+D3), FA, or vitamin B12 greater than 0 micrograms within the past 30 days (i.e., any nutrient came from supplements rather than just food), it was classified as “using supplements”; On the contrary, it was classified as “unused” (Centers for Disease Control and Prevention). (8) Depression: According to the NHANES Patient Health Questionnaire (PHQ‐9) data, a total score of ≥ 10 was defined as the presence of depression (Varghese et al. 2022). (9) Sleep disorders: The presence of sleep disorders was determined by the questionnaire question “Have you ever been informed of having sleep disorders?” with a “yes” answer.

### 2.5 Statistical Analysis

Data was analyzed by R (V4.4.1) software. The “tableone” package was employed to draw a baseline table in which categorical variables were represented by sample size and proportion and continuous variables by average and standard deviation. Among them, the sample size of categorical variables was not adjusted by weight, and other statistical indicators were adjusted by weight. A weighted logistic regression model was established by utilizing the “survey” package between different serum vitamins (vitamin B12, vitamin D, and FA) and cognitive impairment in the elderly. Since the skewed distribution of vitamins, we performed a logarithmic transformation of all serum vitamin levels.

To assess the interplay among vitamins, Pearson correlation coefficients were calculated among the three serum vitamins. Further grouping was achieved by using a hierarchical clustering method, where Group 1 consisted of vitamin D and FA, and Group 2 consisted of vitamin B12, to avoid multiple collinearity problems. Then, we established the Bayesian Kernel Machine Regression (BKMR) model using the “bkmr” package. BKMR is a semi‐parametric approach that allows kernel functions to model nonlinear and non‐additive exposures to predict the effects of individual mixture components, the overall effects of the mixture components, and potential interactions between the mixture components (Bobb et al. 2015, Menardo et al. 1988). We employed a stratified variable selection method to estimate group posterior inclusion probability (groupPIP) and conditional PIP (condPIP). The former represents the likelihood of a particular group being incorporated into the model, while the latter represents the probability that specific vitamins within that group are included (Du et al. 2024). We applied the BKMR model to investigate the linkage between vitamin‐mixed exposure and cognitive impairment in the elderly, mainly focusing on (1) the overall effect of multivitamin‐combined exposure on cognitive disorder; (2) the effects of changes in individual vitamin levels (other vitamins maintained at the 50th percentile) on cognitive disorders; and (3) the combined effect of two vitamins, that is, the effect of one vitamin at 25%, 50%, and 75%, respectively, on cognitive impairment (the other vitamins remained at the 50% level).

A Weighted Quantile Sum (WQS) model was applied to examine the combined effects of multivitamin mixtures on cognitive impairment in the elderly. In the WQS model, all vitamins were considered and limited to have the same relevant orientation to the results, and the weight of each vitamin reflected its contribution to the overall effect (Shi et al. 2023). *p *< 0.05 in this study was considered statistically significant.

In addition, we conducted a series of sensitivity analyses and subgroup analyses to test the robustness of the results and explore specific associations in depth. First, to evaluate the potential confounding effects of comorbidities related to cognitive impairment, such as depression and sleep disorders, we included them as additional covariates in the logistic regression model for sensitivity analysis. To investigate the relationship between high vitamin B12 levels and cognitive impairment, we defined serum vitamin B12 levels > 665 pmol/L as the high B12 state (Kakumani et al. 2024). On this basis, we used a weighted logistic regression model to conduct stratified subgroup analysis for different demographic characteristics, lifestyles, and complications (hypertension, diabetes, BMI grouping, anemia, kidney stones, hyperuricemia, etc.) and calculated the odds ratio (OR) and 95% confidence interval (CI) of high B12 status and cognitive impairment risk in each subgroup.

## 3 Results

### 3.1 Baseline Characteristics

We analyzed 2582 participants, with an average age of 69.08 ± 6.63 years. Participants were grouped according to whether they had cognitive impairment or not. Participants in the cognitively impaired group were older (72.51 ± 6.79 vs. 67.94 ± 6.17, *p *< 0.001), had a higher proportion of males (52.3% vs. 44.3%, *p *= 0.012), had a higher proportion of educational levels of high school or below (64.4% vs. 28.5%, *p *< 0.001), and had a higher proportion of hypertension (85.1% vs 73.7%, *p *< 0.001), diabetes (32.8% vs. 21.5%, *p *= 0.002) and depression (10.4% vs. 5.9%, *p *< 0.001) than those in the normal group. In addition, the HDL‐C (53.33 ± 16.76 vs. 56.28 ± 16.59, *p *= 0.003), total cholesterol (183.20 ± 41.52 vs. 195.59 ± 41.63, *p *< 0.001), vitamin D (79.36 ± 30.60 vs. 83.33 ± 30.86, *p *= 0.005), and the use of vitamin supplements (34.3% vs. 42.4%, *p *= 0.001) of participants were greatly lower in the group with cognitive impairment than the normal group (Table 1).

**TABLE 1:** Baseline characteristics of participants.

Characters	Total	Normal cognition	Cognitive impairment	*p* value
Overall	2582	1632 (75.1)	950 (24.9)	
Age	69.08 (6.63)	67.94 (6.17)	72.51 (6.79)	< 0.001
Gender				0.012
Male	1270 (46.3)	725 (44.3)	545 (52.3)	
Female	1312 (53.7)	907 (55.7)	405 (47.7)	
BMI (kg/m^2^)				0.231
< 25	689 (26.1)	428 (25.3)	261 (28.5)	
25–30	935 (36.9)	580 (36.4)	355 (38.5)	
> 30	958 (37.0)	624 (38.4)	334 (32.9)	
Physical activity				< 0.001
None	487 (23.0)	378 (27.2)	109 (10.3)	
Moderate	1038 (40.1)	690 (41.4)	348 (36.3)	
Intense	1057 (36.9)	564 (31.4)	493 (53.5)	
Alcohol				< 0.001
No	804 (26.7)	462 (23.8)	342 (35.7)	
Yes	1778 (73.3)	1170 (76.2)	608 (64.3)	
Smoke				0.213
Never	1271 (49.8)	826 (50.5)	445 (47.7)	
Past	975 (39.2)	615 (39.1)	360 (39.2)	
Now	336 (11.1)	191 (10.4)	145 (13.1)	
Education				< 0.001
Less than high school	282 (5.5)	47 (1.7)	235 (16.8)	
High school or equivalent	958 (32.0)	523 (26.8)	435 (47.6)	
College or above	1342 (62.6)	1062 (71.5)	280 (35.6)	
Hypertension				< 0.001
No	538 (23.4)	381 (26.3)	157 (14.9)	
Yes	2044 (76.6)	1251 (73.7)	793 (85.1)	
Diabetes				0.002
No	1807 (75.7)	1198 (78.5)	609 (67.2)	
Yes	775 (24.3)	434 (21.5)	341 (32.8)	
HDL‐C (mg/dL)	55.55 (16.68)	56.28 (16.59)	53.33 (16.76)	0.003
Total cholesterol (mg/dL)	192.50 (41.94)	195.59 (41.63)	183.20 (41.52)	< 0.001
Vitamin D (nmol/L)	82.34 (30.84)	83.33 (30.86)	79.36 (30.60)	0.005
Vitamin B12 (pmol/L)	512.41 (512.35)	508.70 (522.24)	523.61 (481.52)	0.571
Folic acid (nmol/L)	57.32 (35.36)	57.14 (34.26)	57.86 (38.50)	0.706
Vitamin supplements				0.001
No	1694 (59.6)	1015 (57.6)	679 (65.7)	
Yes	888 (40.4)	617 (42.4)	271 (34.3)	
Depression				< 0.001
No	2334 (93.0)	1508 (94.1)	826 (89.6)	
Yes	232 (7.0)	111 (5.9)	121 (10.4)	
Sleep disorders				0.565
No	2259 (88.0)	1420 (87.6)	839 (89.1)	
Yes	307 (12.0)	199 (12.4)	108 (10.9)	

Abbreviations: BMI, body mass index; HDL‐C, high‐density lipoprotein cholesterol.

### 3.2 Linkage Between Serum Vitamins and Cognitive Impairment in the Elderly

A weighted logistic regression model was used to study the linkage between different serum vitamins and cognitive impairment in the elderly. The model adjusted for all covariates showed that vitamin D (OR = 0.695, 95% CI: 0.534‐0.905, *p *= 0.003) and FA (OR = 0.777, 95% CI: 0.604‐0.999, *p *= 0.034) were negatively linked with cognitive impairment in the elderly. No pronounced relationship was detected between vitamin B12 and cognitive impairment in the elderly (Table 2).

**TABLE 2:** Associations between serum vitamin levels and cognitive impairment in the elderly.

Exposures	Crude model		Adjusted model	
	OR (95% CI)	*p*	OR (95% CI)	*p*
Vitamin B12	1.049 (0.891–1.235)	0.551	1.117 (0.917–1.360)	0.234
Vitamin D	0.716 (0.580–0.884)	0.001	0.695 (0.534–0.905)	0.003
Folic acid	0.954 (0.789–1.153)	0.609	0.777 (0.604–0.999)	0.034

*Note*: The crude model was unadjusted for confounding factors. The adjusted model was adjusted for sex, age, BMI, smoking status, alcohol consumption, education level, hypertension, diabetes, physical activity, total cholesterol, and high‐density lipoprotein cholesterol.

Given that baseline characteristics showed significant differences in vitamin supplement use between cognitively normal and impaired groups, we conducted a sensitivity analysis and included vitamin supplement use as an additional covariate in the model for correction. As shown in Table S1, after comprehensive adjustment, the negative correlation between serum vitamin D and cognitive impairment remained significant (OR = 0.727, 95% CI: 0.551‐0.959, *p *= 0.014).

Considering that depression and sleep disorders are both adverse consequences of cognitive impairment and potential confounding factors, we conducted a sensitivity analysis and included both in the model for correction. The results showed that the negative correlation between serum vitamin D and cognitive impairment remained significant in a sample of 2566 participants (OR = 0.731, 95% CI: 0.550‐0.972, *p *= 0.018), further confirming the robustness of this association (Table S2).

### 3.3 BKMR Analysis

**FIGURE 2:**
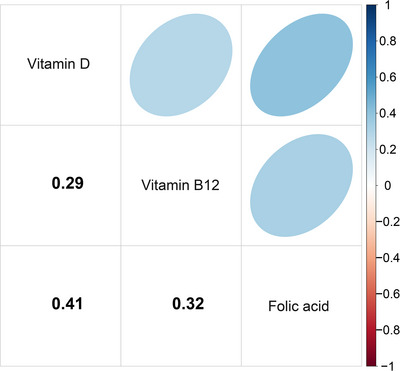
Pearson correlation analysis among serum vitamins. All correlation coefficients were statistically significant (*p *< 0.001). The specific *r* values are: Vitamin D and FA, *r* = 0.41; Vitamin D and vitamin B12, *r* = 0.29; FA and Vitamin B12, *r* = 0.32.

We calculated the correlation between the three serum vitamins, in which the correlation coefficient between vitamin D and FA was 0.41 (*p *< 0.001), and the correlation between the two was relatively high (Figure 2, Table S3). The serum vitamins were divided into two groups by hierarchical cluster analysis according to correlation coefficients. Group PIP and condPIP based on the BKMR model showed that Group 1 (vitamin D and FA) had the highest groupPIP of 0.946, indicating that this group of vitamins was of high importance in predicting the risk of cognitive impairment in the elderly. After the control of the other variables, FA in Group 1 had the highest condPIP of 0.715, indicating its importance in the influence of cognitive impairment in the elderly. In contrast, Group 2 (vitamin B12) had a lower groupPIP (0.456), indicating that vitamin B12 was less important in models that predict the risk of cognitive impairment in older adults (Table 3).

**TABLE 3:** GroupPIP and condPIP based on the BKMR model.

Exposures	Group	Cognitive impairment	
		groupPIP	condPIP
Vitamin D	1	0.946	0.285
Folic acid	1	0.946	0.715
Vitamin B12	2	0.456	1.000

*Note*: The model was adjusted for sex, age, BMI, smoking status, alcohol consumption, education level, hypertension, diabetes, physical activity, total cholesterol, and high‐density lipoprotein cholesterol.

Abbreviations: condPIP, conditional posterior inclusion probability.; groupPIP, group posterior inclusion probability.

**FIGURE 3:**
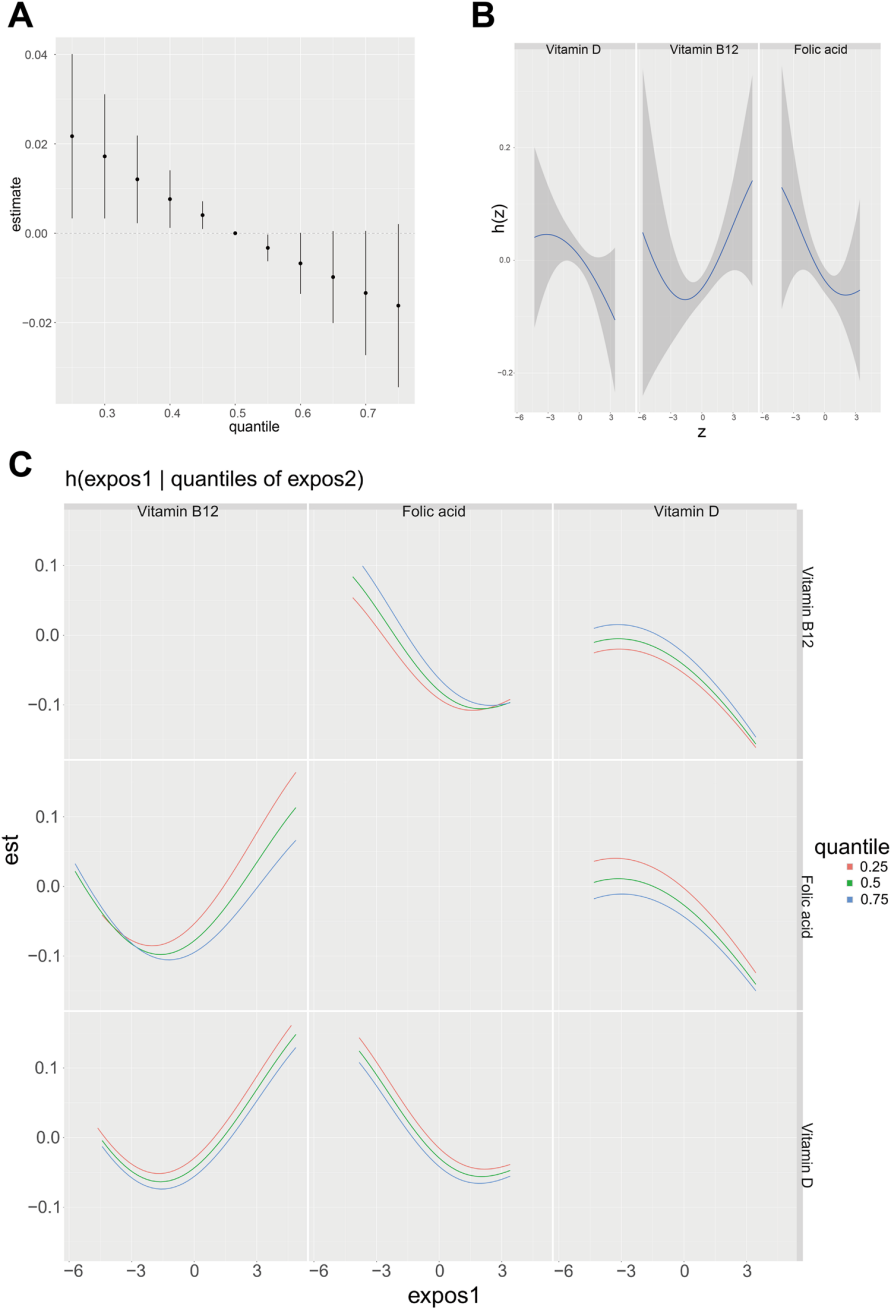
Associations between serum vitamin levels and risk of cognitive impairment in older adults based on BKMR models. (A) The overall effect of the three serum vitamins on cognitive impairment risk. (B) Univariate exposure‐response relationship between individual vitamins and cognitive impairment risk. h(z) represents the strength of the association between vitamins and cognitive impairment risk. (C) Bivariate exposure‐response relationship between vitamins and cognitive impairment risk.

We assessed the overall effects of three serum vitamins on the risk of cognitive impairment in older people. Adjusted covariates showed that serum vitamin levels below the 50th percentile were linked with a considerably elevated risk of cognitive impairment in the elderly (Figure 3A), as compared with mixed vitamin levels at the 50th percentile. The exposure‐response relationship of each serum vitamin level was further investigated. The results showed that vitamin D and FA were negatively correlated with cognitive disorders, while vitamin B12 had a U‐shaped association with cognitive disorders (Figure 3B). We also explored the interaction between the three serum vitamins, with only a potential interaction observed between vitamin B12 and FA. No apparent interaction was detected among the other vitamins (Figure 3C).

### 3.4 WQS Analysis

**FIGURE 4:**
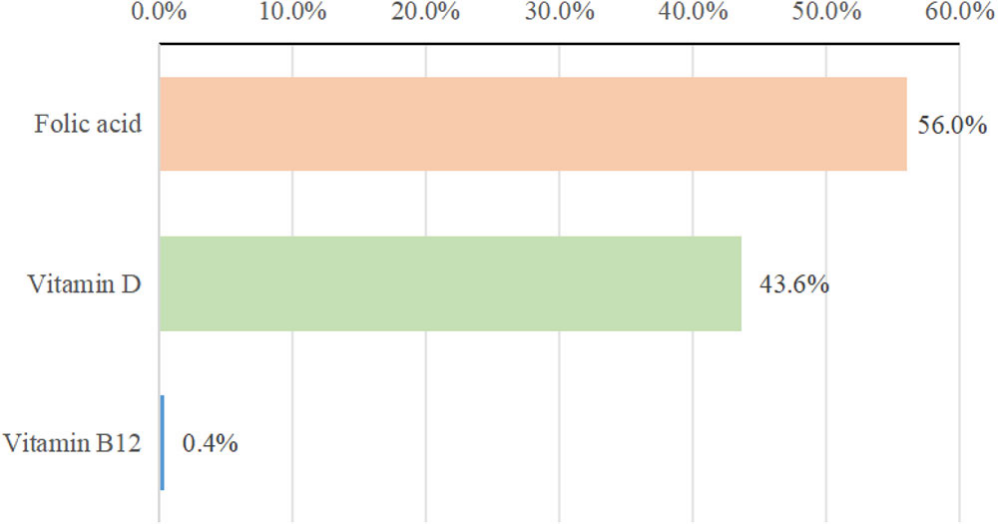
Relative contributions of three serum vitamins to the overall effect evaluated by the weighted quantile sum model.

Figure 4 shows the extent to which three serum vitamins contribute to reducing the risk of cognitive impairment in the elderly. Among them, FA contributed the most to lowering the risk of cognitive impairment in the elderly, accounting for 56.0%, followed by vitamin D (43.6%). The contribution of vitamin B12 was small, only 0.4%, indicating that its role in lowering the risk of cognitive impairment in the elderly was relatively limited.

### 3.5 Subgroup Analysis

To elucidate the correlation between a high level of vitamin B12 and cognitive impairment in the U‐shaped relationship, we defined a high B12 state (*n* = 492) based on the literature threshold (> 665 pmol/L) and conducted stratified analysis. The results showed that the association between high B12 status and the risk of cognitive impairment was not evenly distributed but highly concentrated in specific subgroups (Table S4). Among participants with a history of kidney stones, the association between high B12 status and the risk of cognitive impairment was strongest (OR = 3.314, 95% CI: 1.358–8.088, *p *= 0.004). In addition, among participants with a BMI of 25–30 (OR = 2.402, 95% CI: 1.318–4.376, *p *= 0.002), hyperuricemia (OR = 1.665, 95% CI: 0.981–2.828, *p *= 0.039), or hypertension (OR = 1.332, 95% CI: 1.035–1.714, *p *= 0.015), high B12 status also significantly increased the risk of cognitive impairment. In most subgroups without these comorbidities, no significant association was observed.

## 4 Discussion

Based on the NHANES database, we systematically explored the relationship between three key serum vitamins (vitamin D, FA, and vitamin B12) and their mixtures with cognitive impairment in the elderly. The results showed that serum vitamin D and FA levels were significantly inversely linked with the risk of cognitive disorders, while vitamin B12 had a U‐shaped association with the risk of cognitive disorders. Importantly, through the BKMR and WQS models, we quantified for the first time the core contribution of FA in protective vitamin blends (56.0%) in the context of FA enhancement and revealed the close association between high vitamin B12 and specific metabolic comorbidities (such as kidney stones and hypertension) through subgroup analysis. The study provides a new explanatory perspective for understanding the U‐shaped relationship between high vitamin B12 and comorbidities. Our work not only clarifies the importance of vitamin D and FA but also deepens the understanding of the complex role of vitamin B12, providing key evidence for precise nutritional intervention for cognitive impairment in the elderly population.

A particularly important finding of this study is that serum FA levels are still significantly negatively correlated with the risk of cognitive impairment in the US population who have implemented a nationwide FA strengthening policy. Since 1998, the US has implemented a mandatory FA‐strengthening policy, significantly increasing the population's FA levels and making FA deficiency rare (Hildebrand et al. 2021). The average serum FA level of participants in this study was 57.32 nmol/L, which is much higher than the typical level in countries that have not implemented the strengthening policy (about 17 nmol/L) (Quinn et al. 2024), and is within the expected range under the strengthening policy. Therefore, our research findings reveal a key message: in populations where FA deficiency has been largely eliminated through public policies, there still exists a dose‐response relationship of public health significance between FA levels and cognitive function. This finding strongly suggests that the protective effect of FA on the nervous system may not be limited to preventing clinical deficiencies but is equally crucial for promoting optimal cognitive health in elderly adults. Although the current strengthening policies have successfully improved the baseline level, the average level of the subjects in this study is still below the recommended median target for serum FA for the most effective prevention of neural tube defects (44 ng/mL, approximately 99.7 nmol/L) (Wald et al. 2025). Therefore, further focusing on and optimizing the FA nutrition status of the elderly population, while strengthening the existing achievements of policies, may be a valuable direction for preventing cognitive decline in the future.

FA is a vitamin that is instrumental for health at all ages and is especially essential for central nervous system function (Ebara 2017). The conclusion of this study is in line with the findings of multiple cutting‐edge observational studies and intervention trials. A national geriatric study from Israel showed that the presence of serum FA deficiency was linked with a higher risk of dementia compared with the absence of serum FA deficiency (Rotstein et al. 2022). According to another longitudinal study, high serum FA levels were linked with a lower risk of mild cognitive disorder, with the highest quartile array having a 34% lower risk of cognitive impairment than the lowest quartile array of FA (Fu et al. 2022). In a study of vascular dementia and dementia with Alzheimer's disease, 93% of patients with Alzheimer's disease and 91.3% of patients with vascular dementia had low or very low FA levels, compared with 11.3% in the healthy population (Moretti et al. 2017). Additionally, patients with mild cognitive impairment have significantly improved cognitive function and biochemical indicators such as serum homocysteine after six months of FA supplementation (Ma et al. 2016). These findings collectively strengthen the credibility of the results of this study and highlight the central role of FA in delaying cognitive decline in elderly adults.

FA may contribute to cognitive health through a variety of mechanisms. FA deficiency can lead to hyperhomocysteinemia (Kaye et al. 2020). Homocysteine induces DNA damage responses that trigger neuronal apoptosis and increase the sensitivity of hippocampal neurons to excitatory toxicity and oxidative damage, promoting the onset and progression of neurodegenerative diseases (Kruman et al. 2000). Hyperhomocysteine status is also closely associated with endothelial dysfunction and atherosclerosis, leading to cerebral microvascular lesions and insufficient cerebral blood supply and increasing the risk of stroke and vascular dementia (Jakubowski and Witucki 2025, Song et al. 2022). FA supplementation may improve cognitive function by lowering inflammatory markers (H. Chen et al. 2016). In addition, it protects neuronal integrity and improves memory function by increasing superoxide dismutase, catalase activity, and glutathione content in brain tissue of older rats, thus lowering lipid peroxidation levels, alleviating oxidative stress damage, and protecting the integrity of neurons and improving their memory function (Singh et al. 2011).

Vitamin D is an indispensable nutrient for the human body, including two forms: vitamin D2 and vitamin D3. Among them, vitamin D2 is mostly found in plant foods, while vitamin D3 is present in animal foods like fatty fish and fish liver oils in herring and mackerel, as well as in human skin self‐synthesis after exposure to sunlight (Delrue and Speeckaert 2023, Brustad and Meyer 2023). In the body, vitamin D is converted to 25(OH)‐vitamin D by hydroxylation in the liver, which is the main stored form of vitamin D in the body (Henry 2011). We observed that higher vitamin D levels were linked with a reduced risk of cognitive disorders, which is consistent with previous research results. A previous study has shown that people with 25(OH)‐vitamin D levels below 50 nmol/L have a significant likelihood of developing cognitive impairment compared to those with sufficient 25(OH)‐vitamin D (≥ 75 nmol/L) (Llewellyn et al. 2011). In a prospective study, the Mini‐Mental State Examinations (MMSE) scores of patients with severe 25(OH)‐vitamin D deficiencies (< 25 nmol/L) decreased by 0.3 more per year than those with vitamin D sufficiency after multivariate adjustment (Llewellyn et al. 2010). In addition, a study that included 299 patients over the age of 65 revealed that these patients had levels of 25(OH)‐vitamin D below 30 ng/mL on average, and all of them had vitamin D deficiency. MMSE and Montreal Cognitive Assessment scores were lower, and the risk of cognitive dysfunction was higher in patients with severe vitamin D deficiency (< 10 ng/mL) (Lu et al. 2021).

The explanation for vitamin D to lower the risk of cognitive impairment may involve many aspects. First, vitamin D receptors are widely distributed in multiple regions of the brain, including the temporal lobes, cingulate gyrus, hypothalamus, and different regions of the hippocampus. The activation of vitamin D receptors prevents neurological dysfunction and neuronal death and thus plays a neuroprotective role (Eyles et al. 2005, Yuan et al. 2018). Secondly, vitamin D reduced levels of interleukin‐6 (IL‐6) and the oxidized stress marker malondialdehyde to protect against inflammation‐induced cognitive dysfunction (Mokhtari‐Zaer et al. 2020). Furthermore, vitamin D slows the decline in synaptic function and cognitive ability during brain aging by up‐regulating the expression of synaptic protein and neurotransmitter‐related genes, boosting the recirculation of synaptic follicles and neurotransmitter release, and targeting calcium regulatory pathways in the hippocampus (Mokhtari‐Zaer et al. 2020).

This study revealed a U‐shaped relationship between vitamin B12 and the risk of cognitive impairment, where low or high levels of vitamin B12 may increase the risk of cognitive impairment. This finding is consistent with previous studies, most of which have confirmed the adverse effects of vitamin B12 deficiency on cognitive function (Nalder et al. 2021, Vinueza Veloz et al. 2022, Xu et al. 2022). However, regarding the harm of high levels of B12, although research suggests an inverted U‐shaped association with cognitive function (Ding et al. 2022), its underlying mechanism is still unclear. Of particular importance, the subgroup analysis in this study provides key clues to elucidate the potential causes of excessive B12 levels. We found that the harmful association of high B12 levels (> 665 pmol/L) is not widespread but highly concentrated in individuals with specific metabolic and renal comorbidities, such as kidney stones, hypertension, overweight, and hyperuricemia. This model strongly suggests that the observed high B12 risk may not be a direct neurotoxic effect of vitamin B12 itself, but rather a biomarker of potential health status. For example, impaired kidney function (indirectly reflected by kidney stones and hyperuricemia) may affect the metabolism and clearance of vitamin B12, leading to its abnormal accumulation in the body (Wu and Wang 2022, Nielsen et al. 2022). Meanwhile, these pathological states themselves directly increase the risk of cognitive impairment (Shi et al. 2018, Capasso et al. 2025). Therefore, the association between high B12 levels and cognitive impairment is likely to reflect the combined effects of these underlying metabolic disorders and renal dysfunction that are not fully controlled.

In addition, our study observed a potential interaction between vitamin B12 and FA. Previous studies have found that in the absence of vitamin B12, high levels of FA can further impair the activity of vitamin B12‐dependent enzymes such as methionine synthase and methylmalonate CoA mutase, leading to elevated levels of circulating homocysteine and methylmalonate. This interaction may exacerbate neurological damage and cognitive decline (Selhub et al. 2009, Selhub et al. 2022). This finding, together with the U‐shaped relationship mentioned above, suggests that in clinical practice, it is crucial for elderly people to maintain appropriate levels of vitamin B12. In addition to being vigilant about the vitamin B12 deficiency, we should also regard abnormally elevated B12 levels as a warning signal for individuals with metabolic or kidney problems.

In summary, the results of this study provide important directions for precise nutritional interventions for cognitive impairment in the elderly population. First, in areas where FA enhancement has been implemented, public health strategies should shift from preventing deficiency to optimizing levels to obtain cognitive health benefits. Elderly people should be encouraged to consume natural foods such as green leafy vegetables and animal liver and take necessary supplements under monitoring in order to maintain an ideal FA nutritional status. Secondly, the management of vitamin B12 should be flexible and specific, with clinical practice taken in conjunction with individual health conditions. For elderly people with metabolic problems such as hypertension, overweight, kidney stones, or hyperuricemia, their abnormally elevated vitamin B12 levels should be considered as a warning signal that requires a comprehensive assessment of health risks. Meanwhile, vitamin B12 levels should be in an appropriate range and become balanced with FA supplementation, which is crucial for synergistically protecting cognitive function. In addition, adhering to the intake of fatty fish and moderate sun exposure to increase vitamin D levels is still an essential basic measure.

However, this research is subject to certain constraints. The cross‐sectional nature of the study prevents the causality analysis from inferring the causal relationship between serum vitamins and cognitive impairment. Secondly, a one‐time serum vitamin assessment may not capture chronic nutritional status. Thirdly, the NHANES 2011–2014 cycle used in this study lacks systematic data on homocysteine, making it impossible to evaluate the impact of this important mediating factor in the model. Finally, the results are based on the American population, and the universality of the findings of the study needs to be further verified in other ethnic or regional populations. Additional research is warranted to elucidate the long‐term relationship between vitamin levels and cognitive function changes in prospective cohorts containing complete homocysteine data, trying to clarify the optimal nutritional intervention strategies for specific populations.

## 5 Conclusion

Maintaining sufficient levels of serum vitamin D and FA is key to protecting cognitive health in the elderly. There is a U‐shaped association between vitamin B12 and cognitive impairment, suggesting that vitamin B12 should be maintained within an appropriate range. These findings provide important insights for public health practice: in areas where FA supplementation has been implemented, the focus of nutritional interventions should shift from preventing deficiency to optimizing levels to obtain cognitive health benefits. Meanwhile, the management of vitamin B12 should be more targeted, with particular attention paid to its significance as a potential health risk marker in individuals with metabolic complications. Therefore, developing personalized nutrition strategies that combine diet, sunlight, and necessary supplements, and implementing risk stratification management for vitamin B12, are crucial for effectively reducing the risk of cognitive impairment in the elderly population.

## Author Contributions

All authors contributed to data analysis, drafting and revising the article, gave final approval of the version to be published, and agreed to be accountable for all aspects of the work.

## Funding

This study was supported by the National Natural Science Foundation of China Project, No. 82374166, and the Liaoning Provincial Department of Education Basic Research Project (General Program), No. LJKMZ20221317.

## Ethics Statement

The authors have nothing to report.

## Conflicts of Interest

The authors declare no conflicts of interest.

## Supporting information




**Supplementary Table**: brb371181‐sup‐0001‐TableS1.docx

**Supplementary Table**: brb371181‐sup‐0002‐TableS2.docx

**Supplementary Table**: brb371181‐sup‐0003‐TableS3.docx

**Supplementary Table**: brb371181‐sup‐0004‐TableS4.docx

## Data Availability

The data that support the findings of this study are available from the corresponding author upon reasonable request.
