# Supplementary material for: Relationship Between Serum Vitamins and Cognitive Impairment in the Elderly: A Study Based on the NHANES Database
Source: Brain Behav. 2026 Jan 13;16(1):e71181. doi: 10.1002/brb3.71181 (PMC12796845; doi:10.1002/brb3.71181)
Supplement: Supplementary file 1 — Supplementary Table: brb371181‐sup‐0001‐TableS1.docx [file BRB3-16-e71181-s001.docx]

**Table S1:Results of the Sensitivity Analysis (Adjusted for vitamin supplement use)**

| **Exposures** | **Adjusted model** | |
| --- | --- | --- |
|  | **OR (95%CI)** | **P** |
| Vitamin B12 | 1.182 (0.947-1.476) | 0.108 |
| Vitamin D | 0.727 (0.551-0.959) | 0.014 |
| Folic acid | 0.803 (0.602-1.070) | 0.103 |

Note: This model has adjusted for gender, age, BMI, smoking, drinking, educational level, hypertension, diabetes, physical activity, total cholesterol, high-density lipoprotein cholesterol, and vitamin supplement use.
