# Supplementary material for: Relationship Between Serum Vitamins and Cognitive Impairment in the Elderly: A Study Based on the NHANES Database
Source: Brain Behav. 2026 Jan 13;16(1):e71181. doi: 10.1002/brb3.71181 (PMC12796845; doi:10.1002/brb3.71181)
Supplement: Supplementary file 2 — Supplementary Table: brb371181‐sup‐0002‐TableS2.docx [file BRB3-16-e71181-s002.docx]

**Table S2:The Relationship between Serum Vitamins and Cognitive Impairment in the Elderly**

| **Exposures** | **Adjusted model** | |
| --- | --- | --- |
|  | **OR (95%CI)** | **P** |
| Vitamin B12 | 1.169 (0.887-1.540) | 0.224 |
| Vitamin D | 0.731 (0.550-0.972) | 0.018 |
| Folic acid | 0.787 (0.594-1.043) | 0.068 |

Note: Due to the skewed distribution of serum vitamins, the above analysis performed a logarithmic transformation on all vitamin levels.

Adjusted model: Adjusted for gender, age, BMI, smoking, drinking, education level, hypertension, diabetes, physical activity, total cholesterol, high-density lipoprotein cholesterol, depression, and sleep disorders.
