# Supplementary material for: Relationship Between Serum Vitamins and Cognitive Impairment in the Elderly: A Study Based on the NHANES Database
Source: Brain Behav. 2026 Jan 13;16(1):e71181. doi: 10.1002/brb3.71181 (PMC12796845; doi:10.1002/brb3.71181)
Supplement: Supplementary file 3 — Supplementary Table: brb371181‐sup‐0003‐TableS3.docx [file BRB3-16-e71181-s004.docx]

**Table S3:Pearson correlation coefficient (r value) and the corresponding P value**

| Vitamins | Vitamin D and folic acid | Vitamin D and vitamin B12 | Folic acid and vitamin B12 |
| --- | --- | --- | --- |
| r | 0.41 | 0.29 | 0.32 |
| P-value | <0.001 | <0.001 | <0.001 |
