# Supplementary material for: Relationship Between Serum Vitamins and Cognitive Impairment in the Elderly: A Study Based on the NHANES Database
Source: Brain Behav. 2026 Jan 13;16(1):e71181. doi: 10.1002/brb3.71181 (PMC12796845; doi:10.1002/brb3.71181)
Supplement: Supplementary file 4 — Supplementary Table: brb371181‐sup‐0004‐TableS4.docx [file BRB3-16-e71181-s003.docx]

**Table S4. Association between high vitamin B12 status and cognitive impairment across subgroups of older adults**

| **Characters** | **Adjusted Model** | |
| --- | --- | --- |
|  | **OR (95% CI)** | **p** |
|  |  |  |
| **Alcohol** |  |  |
| No | 1.495 (0.831-2.690) | 0.142 |
| Yes | 1.345 (0.925-1.957) | 0.090 |
| **Smoke** |  |  |
| Never | 1.383 (0.935-2.044) | 0.077 |
| Past | 1.315 (0.812-2.129) | 0.226 |
| Now | 1.807 (0.466-7.015) | 0.352 |
| **Hypertension** |  |  |
| No | 1.358 (0.634-2.907) | 0.389 |
| Yes | 1.332 (1.035-1.714) | 0.015 |
| **Diabetes** |  |  |
| No | 1.426 (0.951-2.136) | 0.060 |
| Yes | 1.288 (0.769-2.159) | 0.293 |
| **BMI** |  |  |
| <25 | 1.127 (0.666-1.907) | 0.628 |
| 25-30 | 2.402 (1.318-4.376) | 0.002 |
| >30 | 0.839 (0.479-1.470) | 0.505 |
| **Anemia** |  |  |
| No | 1.400 (1.001-1.956) | 0.031 |
| Yes | 1.409 (0.632-3.140) | 0.356 |
| **Kidney stone** |  |  |
| No | 1.255 (0.907-1.737) | 0.133 |
| Yes | 3.314 (1.358-8.088) | 0.004 |
| **Hyperuricemia** |  |  |
| No | 1.350 (0.887-2.056) | 0.125 |
| Yes | 1.665 (0.981-2.828) | 0.039 |

Adjusted model: Adjusted for gender, age, BMI, smoking, drinking, education level, hypertension, diabetes, physical activity, total cholesterol, high-density lipoprotein cholesterol, anemia, kidney stones, and hyperuricemia.
